# Differential Kinetics in Alteration and Recovery of Cognitive Processes from a Chronic Sleep Restriction in Young Healthy Men

**DOI:** 10.3389/fnbeh.2016.00095

**Published:** 2016-05-23

**Authors:** Arnaud Rabat, Danielle Gomez-Merino, Laura Roca-Paixao, Clément Bougard, Pascal Van Beers, Garance Dispersyn, Mathias Guillard, Cyprien Bourrilhon, Catherine Drogou, Pierrick J. Arnal, Fabien Sauvet, Damien Leger, Mounir Chennaoui

**Affiliations:** ^1^Fatigue and Vigilance Unit, Department of Neurosciences and Operational Constraints, Armed Forces Biomedical Research Institute (IRBA)Brétigny-sur-Orge, France; ^2^VIFASOM team (EA 7330), University of Paris 5 DescartesParis, France; ^3^University of Paris 11Orsay, France; ^4^Department of Operational Environments, Armed Forces Biomedical Research Institute (IRBA)Brétigny-sur-Orge, France; ^5^Alertness and Sleep Center, Hôtel Dieu de Paris, Public Assistance of Paris Hospitals, University of Paris 5 DescartesParis, France

**Keywords:** chronic sleep debt, sustained attention, executive processes, time of day, recovery, age, hormones

## Abstract

Chronic sleep restriction (CSR) induces neurobehavioral deficits in young and healthy people with a morning failure of sustained attention process. Testing both the kinetic of failure and recovery of different cognitive processes (i.e., attention, executive) under CSR and their potential links with subject’s capacities (stay awake, baseline performance, age) and with some biological markers of stress and anabolism would be useful in order to understand the role of sleep debt on human behavior. Twelve healthy subjects spent 14 days in laboratory with 2 baseline days (B1 and B2, 8 h TIB) followed by 7 days of sleep restriction (SR1-SR7, 4 h TIB), 3 sleep recovery days (R1–R3, 8 h TIB) and two more ones 8 days later (R12–R13). Subjective sleepiness (KSS), maintenance of wakefulness latencies (MWT) were evaluated four times a day (10:00, 12:00 a.m. and 2:00, 4:00 p.m.) and cognitive tests were realized at morning (8:30 a.m.) and evening (6:30 p.m.) sessions during B2, SR1, SR4, SR7, R2, R3 and R13. Saliva (B2, SR7, R2, R13) and blood (B1, SR6, R1, R12) samples were collected in the morning. Cognitive processes were differently impaired and recovered with a more rapid kinetic for sustained attention process. Besides, a significant time of day effect was only evidenced for sustained attention failures that seemed to be related to subject’s age and their morning capacity to stay awake. Executive processes were equally disturbed/recovered during the day and this failure/recovery process seemed to be mainly related to baseline subject’s performance and to their capacity to stay awake. Morning concentrations of testosterone, cortisol and α-amylase were significantly decreased at SR6-SR7, but were either and respectively early (R1), tardily (after R2) and not at all (R13) recovered. All these results suggest a differential deleterious and restorative effect of CSR on cognition through biological changes of the stress pathway and subject’s capacity (ClinicalTrials-NCT01989741).

## Introduction

Sleep, a homeostatic-controlled mechanism, is essential to maintain body and brain efficiency as shown by the well-known consequences of its deprivation (body weight increase, difficulty in concentrating, emotional liability, etc.) that might lead to medical problems and/or to vehicle and industrial accidents (Gander et al., 2005; Cirelli and Tononi, 2008; Goel et al., 2009; Horne, 2011, 2012; Horne and Rumbold, 2015). Based on the Presidential Report of the Challenger accident which had noted that “*working excessive hours, whilst admirable, raises serious questions when it jeopardizes job performance, particularly when critical management decisions are at stake*” (NASA, 1986), it seems reasonable to consider that human errors may not always simply be due to sleepiness, but to more subtle effects on brain that will affect decision-making relevant to the world of work (Horne, 2012).

Indeed, systematically prolonged wakefulness or total sleep deprivation (TSD) situations accompanied by a significant reduction in the latency to sleep onset and/or an increase in the number of involuntary microsleeps (Durmer and Dinges, 2005; Goel et al., 2009; Arnal et al., 2015) is also associated with detrimental effects on various cognitive domains (see reviewed in Chee and Chuah, 2008; Killgore, 2010). For instance, Drummond and colleagues have shown, with a Go-NoGo task (executive process with response inhibition), that subjects submitted to two nights of TSD could initiate a response when appropriate (although somewhat slower than usual) but were no more able to withhold an inappropriate (“*NoGo*”) one (Drummond et al., 2006). In this study, performance on all outcome variables returned to baseline levels after a single night of recovery sleep. Performance deficits have been largely described both in non executive tasks such as psychomotor vigilance test (PVT) that specifically measures sustained attention processes (Lim and Dinges, 2008; Horne, 2010, 2011; Arnal et al., 2015) and in executive ones such as inhibition with Go-NoGo (Drummond et al., 2006), working memory with n-back or Sternberg tasks (Choo et al., 2005; Chee et al., 2006; Tucker et al., 2010) and decision-making with risk (Killgore et al., 2006; McKenna et al., 2007) or dilemma (Killgore et al., 2007a; for review see Chee and Chuah, 2008; Killgore, 2010; Lim and Dinges, 2010; Horne, 2012). TSD is responsible for a significant reduction of cerebral metabolism in regions such as the thalamus, basal ganglia and cortex (Wu et al., 1991; Thomas et al., 2000) for review see Ma et al. (2015) and behavioral deficits (e.g., working memory) observed under such situations seem to be related to a metabolic reduction of prefrontal, parietal and thalamic regions (Choo et al., 2005; Mu et al., 2005b; Chee et al., 2006). Interestingly, with accumulated time awake, sleep pressure increases sleepiness and sustained attention performance is still possible but at the cost of decreasing speed and increasing the number of lapses in PVT, leading to greater intra- and inter-variability (Doran et al., 2001; Van Dongen et al., 2004, 2012). Some recent studies have pointed out the fact that TSD amplified individual differences in sustained attention performances that already existed, to a lesser extent, during baseline hours (Chua et al., 2014a,b; Yeo et al., 2015). Since individual differences in working memory deficits (Mu et al., 2005a; Chee et al., 2006) and no significant interference and facilitation in sensori-motor decision-making (Simon task) have been reported in subjects submitted to TSD (Bratzke et al., 2012), some authors have called into question the claim that sleep loss primarily degrades executive functions and reasoning (Lim and Dinges, 2010; Horne, 2012; Basner et al., 2013; Jackson et al., 2013). These authors are rather in favor of an indirect effect of sleep loss on these high-order cognitive processes through deficits of alertness and sustained attention.

From an ecological point of view, TSD situations are less realistic than chronic sleep restriction (CSR) ones. Indeed epidemiological studies have revealed a higher frequency of workers in chronic sleep debt (i.e., habitual short sleep duration) than totally sleep deprived (Krueger and Friedman, 2009) concomitantly with excessive sleepiness (Drake et al., 2004), medical errors (Barger et al., 2006; Czeisler, 2009; Rothschild et al., 2009; Reed et al., 2010); and more recently an increased risk of obesity (Knutson and Van Cauter, 2008; Kobayashi et al., 2012, 2013) and diabetes (Chao et al., 2011). Much information concerning cognition capacities of these professional workers has been obtained from sleep disruptions in safety critical environments, such as health care (Landrigan, 2005; Barger et al., 2006; Reed et al., 2010) and aviation (Caldwell and Caldwell, 2005; Caldwell, 2005). Laboratory studies inducing insufficient sleep have, for most of them, revealed sustained attention deficits (Belenky et al., 2003; Axelsson et al., 2008; Rupp et al., 2009; Lo et al., 2012; Philip et al., 2012), in a sleep-dose-dependent manner (Belenky et al., 2003; Van Dongen et al., 2003), and for fewer of them executives failures (Van Dongen et al., 2003; Lo et al., 2012). It has also been demonstrated that the endogenous circadian system modulates neurobehavioral deficits across sleep-restriction days during the diurnal portion of the circadian cycle but only for visual performance and sustained attention process (Cohen et al., 2010; Mollicone et al., 2010; Zhou et al., 2011; Lo et al., 2012; Pomplun et al., 2012). To the best of our knowledge, no studies have looked at a differential kinetic of degradation and recovery under CSR for different cognitive processes from simple one (i.e., sensory-motor coordination) to high order ones (e.g., decision-making) going through elementary ones (e.g., attention, inhibition, working memory). This interest is, beyond the scientific benefit, to better appreciate the role of sleep debt on human behavior when an unexpected emergency occurs (e.g., Tchernobyl, Three Mile Island, Fukushima…Rubinstein, 1979; Flin and Stewart, 1996).

Furthermore, influences of time of day on sustained attention have never been demonstrated for other cognitive processes such as executive ones and influences of age (in young subjects) in such cognitive deficits under CSR is not clear (Bliese et al., 2006). Finally, biological mechanisms underlying such cognitive deficits have not been elucidated whereas stress-related activity of the hypothalamo-pituitary (HPA) axis and sympatho-adrenal-medullary (SAM) system and decrease of the anabolic hormone testosterone could be good candidates. Indeed it has been well established that TSD, a stress condition, is responsible for sustained attention deficits and hormonal impairments (Maggio et al., 2013; Arnal et al., 2015, 2016) with a decrease of morning levels of circulating testosterone and cortisol (Arnal et al., 2016). Circadian misalignment/Sleep restriction in humans or REM/TSD in rodents were associated with an increase of adrenal activity and/or a decrease of testosterone levels (Axelsson et al., 2005; Reynolds et al., 2012; Schmid et al., 2012; Zubedat et al., 2013) that could interfere with cognitive functions, including memory (Maggio et al., 2013). Besides, salivary α-amylase (sAA) level is considered as a useful tool for evaluating SAM activity, and was found lowered under chronic psychological stress in association with impairments in cognitive performance (Teixeira et al., 2015).

Aim of this study was thus to assess daytime cognitive performance (through sustained attention, executive and decision-making tasks) along (SR1, 4 and 7) a CSR experiment (4 h TIB during seven nights) and along (R2, R3 and R13) 13 subsequent sleep recovery (8 h TIB) nights. We hypothesized that sleeping 4 h per night for 7 days would differently affect cognition components depending on the time of day, with different morning hormonal responses.

## Materials and Methods

### Subjects

Twelve healthy right-handed male volunteers, aged 29.3 ± 1.37 years (mean ± SEM with an upper and lower bound of 20 and 37 years old), with a normalized BMI (23.8 ± 2.1 kg/m^2^) participated in this laboratory-controlled protocol. The ethics committee of the Hotel Dieu—Ile de France 1 (Paris) and the Agence Nationale pour la Sécurité du Médicament (Drug safety national agency, ANSM) approved the protocol (N°ID RCB: 2012_A00399634), which was conducted according to the principles laid out in the Declaration of Helsinki of 1975, as revised in 2001.

About 50 young male subjects were informed about our experimental protocol and our scientific hypothesis during different meetings. After giving them a written informed consent, all these subjects underwent a detailed medical history and examination in order to exclude subjects with neurological, psychiatric and sleep disorders, drug or alcohol addiction, anxiety and depressive disorders (score under 48 and no higher than 55) and vision problems (colorblind subjects). Other exclusion criteria were: shift-workers, smokers, daily consumers of alcoholic beverages or caffeine (more than 400 mg per day), those taking medication or practice excessive physical activities and subjects older than 40 years old. Subjects with a BMI greater than 27 kg/m^2^, those with an excessive daytime sleepiness (Score at Epworth Sleepiness Scales ≥11; Johns, 1991), bad sleep complaints (Pittsburg sleep quality index “PSQI” > 4; Buysse et al., 1989) or fatigue complaints (Score at French fatigue scale ≥12) and subjects that could not be considered as an intermediate Chronotype (with a score between 39–64) on the Horne and Orstberg questionnaire (Horne and Ostberg, 1976) were also excluded.

Finally we have included 20 male volunteers with a good and homogenous academic level (Equal or Higher than the A-Level). Among these 20 healthy subjects, 12 underwent our experimental protocol with 16 experimental days and 14 nights with a financial compensation.

### Test Instruments and Electrophysiological Recordings

#### Rest/Activity Rhythm

Rest/activity rhythm was evaluated and checked using wrist actigraphy (Actiwatch 7, CamNtech 2008^®^, Cambridge Neurotechnology, Cambridge, Cambridgeshire, UK) and thanks to a sleep–wake diary. Sleep/wake patterns were checked 1 week before the experiment. The mean subject’s total sleep duration was 7.2 ± 0.25 h (mean ± SD).

#### Polysomnography/Sleep Recordings

Polysomnographic (PSG) measures [EEG, EOG, EMG] and EKG (two electrodes placed along the nipple line) were placed accordingly to the international recommendations of the American Academy of Sleep Medicine (AASM; Iber et al., 2007; i.e., 6 electrodes on the scalp at F3–F4, C3–C4, O1–O2 positions; 2 electrodes on the chin and 2 close to the eyes for each lateral movement). All these data were recorded digitally and continuously throughout this experiment in sleep laboratory thanks to a modified version of the Actiwave^®^ system (AW2 and AW45, CamNtech ltd, Upper Pendrill count, Ermine street North, Papworth Everard, Cambridge CB 23304, UK), previously validated for laboratory (Elbaz and Léger, 2008) and ecological situations such as real flights (Sauvet et al., 2014). As recommended by AASM practice parameters, nighttime sleep recordings were analyzed and sleep stages were scored based on the new international sleep classification rules (Iber et al., 2007).

#### Subjective Sleepiness (KSS)

**Figure 1:**
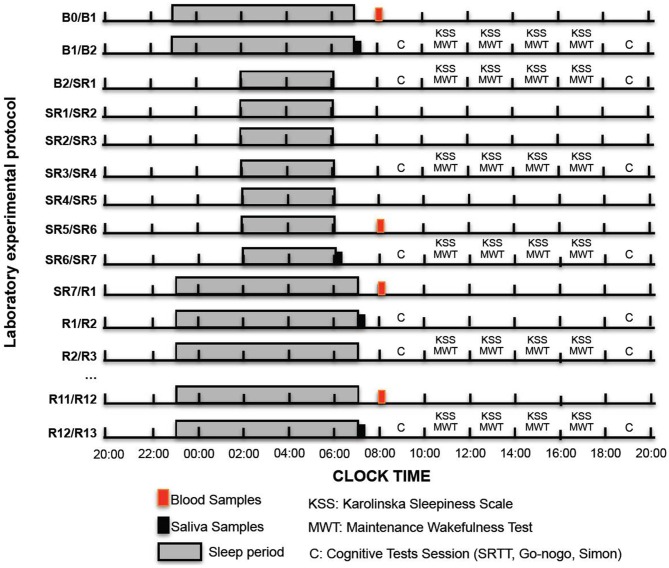
**Experimental design.** We show here the experimental design with sleep time opportunities (gray bars with black lines), blood (red bars) and salivary sampling (black bars), KSS and maintenance of wakefulness sessions (MWT) and cognitive test sessions (C letter) during baseline days (B1 or B2), the 1st, the 4th and the 4th days of sleep restriction (SR1, SR4 and SR7) and during the sleep recovery days (R1 or R2, R3… R13).

Subjective sleepiness was assessed on a single-item scale using the Karolinska Sleepiness Scale (KSS). This scale is 9-point scale based on a self-reported, subjective assessment, of the subject’s level of alertness (Akerstedt and Gillberg, 1990). The dependent measure was the subject’s sleepiness rating. The different levels used for ratings were: 1 = very alert, 3 = alert, 5 = neither alert nor sleepy, 7 = sleepy (but not fighting sleep), 9 = very sleepy (fighting sleep). This scale was completed at 10:00 am, 12:00 am, 2:00 pm and 4:00 pm on B2, SR1, SR4, SR7, R3 and R13 (Figure 1).

#### Capacity to Stay Awake: Maintenance of Wakefulness Test (MWT)

As recommended by AASM practice parameters (Littner et al., 2005) four 40-min Maintenance of Wakefulness Test (MWT) trials were completed at 10:00, 12:00 a.m., 2:00 and 4:00 p.m. respectively during different days of the experimental design (B2, SR1, SR4, SR7, R3, R13; Figure 1). These tests were performed in a sleep laboratory. More precisely, they were completed in darkened rooms according to the AASM recommendations (Littner et al., 2005). These rooms were shielded from external light, and the only light source was positioned behind the subject’s head (0.10–0.13 lux). Room temperature was tuned according to the patient’s comfort level (around 22°C). Experienced sleep technologists and researchers performed the MWT simultaneously for three subjects (thus 2 sessions by MWT trial). Electroencephalogram (F3/A2, F4/A1, C3/A2, C4/A1, O1/A2, O2/A1), electromyogram and electrooculogram were recorded according to the recommendations of the new international sleep scoring classification (Iber et al., 2007). Patients were video monitored during the whole test. Prior to each trial, the patients were asked if they needed other adjustments for comfort. Instructions to the patients were: “*Please sit still and remain awake for as long as possible. Look directly ahead, and do not look directly at the light. Do not stimulate yourself by moving or doing some noise.*” Patients were not allowed to use artificial strategies to stay awake such as slapping the face or singing. Data were recorded and manually analyzed in 30-s epochs using the Embla/Somnologica system (Resmed SAS, ParcTechnologique de Lyon, 292 allée Jacques Monod, 69791 Saint Priest Cedex, France). Sleep latency on the MWT was defined as the first appearance of 1 epoch of any sleep stage (1, 2, 3, 4, or REM). Patients who did not sleep during a trial were assigned a value of 40 min The mean sleep latency of the four MWT trials was then calculated. All recordings were monitored with trained technologists. AASM standard scoring rules, which were updated in 2007, were used to determine sleep latency values (Iber et al., 2007).

#### Visuo-Motor, Sustained Attention, Executive and Decision-Making Tasks

##### Visuo-Motor Pursuit Task

In this task subjects have to, thanks to a joystick, maintain a mobile cursor at the center of the screen between two sets of bars (horizontal and vertical). Amplitudes of lateral and vertical deviations were measured in centimeter from the center (root mean square or RMS) as well as the number of loss of control (when the cursor touch or go through a bar) during all the test session that lasted 3 min.

##### Sustained Attention Task: Simple Reaction Time Task (SRTT)

A 10-min simple reaction time test (SRTT) was performed by subjects on a laptop in order to test their sustained attention capacities (adapted from Gillberg et al., 1994; Sagaspe et al., 2012). A stimulus (a red circle on a black screen) was displayed 100 times on a black screen at randomized (2–7 s) intervals over 10 min. Subjects attended face to the screen of the computer and pressed the left button of the mouse with the preferred index as quickly as possible after the appearance of the visual stimulus. In response to the subject’s button press, the screen displayed the subject’s response latency for 0.5 s, providing trial-by-trial performance feedback. At the end of this 0.5-s interval the display turned off for the remainder of the foreperiod preceding the next stimulus. Foreperiods varied randomly from 2 to 10 s. Dependent measures such as averaged or summed across the 10-min SRTT session were calculated. This measure included average of response latency, its reciprocal mean speed, number of lapses (a response latency exceeding 500 ms), number of anticipation (a response faster than 150 ms) and mean latency for the fastest 10% of all responses.

##### Executive Task (Go-NoGo)

In this executive process task, subject has to either respond or not respond when a stimulus arrived on a screen. After the appearance of a fixation cross in the center of the screen during 500 ms, an arrow appeared in the center of the screen during 1 s (adapted from Sagaspe et al., 2012). Depending on the test instruction, that changes in every session, subjects have to respond as quickly as possible when the arrow pointed out on the right (“go” response) and not to respond when it pointed on the left (“no-go” response). The proportion is always as follows: 67% of “Go” trials and 33% of “No-Go” trials. Subjects have 2 s to respond and their response is directly followed by a new trial in order to determine the cognitive capacity of subjects to consciously inhibit non-relevant automated responses (inhibition process). Time response, omission and commission errors (number of total trials) were the three variables that are taken into account in this task (adapted from Sagaspe et al., 2012).

##### Decision-Making Task (Simon task)

In this 10 min sensori-motor 2-choices reaction time task, subjects have to respond to two different stimuli (adapted from Colzato et al., 2015; Möckel et al., 2015). After appearance of a fixation cross in the center of the laptop screen, a blue or an orange circle appeared either on the right of the left of this cross. If the circle is orange, subjects have to respond with an “*azerty*” keyboard button placed on the left (“Q” letter). If the circle is blue, subjects have to respond with a keyboard button placed on the right (“M” letter). Among all trials, half of them are spatially compatible that is to say that the stimulus arrived on the same side as its corresponding response (i.e., on the left for orange circles and on the right for blue circles) and the other half are spatially incompatible that is to say that the stimulus arrived on the other side as its corresponding response (i.e., on the right for orange circles and on the left for blue circles). Subjects have to respond as quickly and as precisely as possible. Association between color circles and keyboard buttons were randomized between subjects and test sessions. Reaction time, omission and error rates were measured.

### Biological Samples

#### Saliva Samples

A whole saliva sample was collected by passive drool. Salivary samples were collected (non-stimulated samples) immediately after wake-up at 07:00 am on B2 and R13 and at 6:00 am on SR7 (Figure 1). Samples were stored frozen at −80°C until assayed for α-amylase and cortisol concentrations. On the day of testing, all samples were centrifuged at 13,000 g for 4 min. Samples were assayed for α-amylase using a commercial kit (IBL International, Hamburg, Germany), and cortisol was determined using ELISA kit (Salimetrics, State College, PA, USA). Assays were made in duplicate and intra- and inter-assay coefficients of variations (CVs) were 3.0% and 3.0% for cortisol, and 6.7% and 3.6% for α-amylase, respectively. The analytical range of sensitivity for cortisol and α-amylase was 0.33–83 nmol.L^−1^ and 2–400 U.mL^−1^, respectively.

#### Blood Samples

Blood samples were collected via an indwelling venous forearm catheter at 08:00 a.m. during B1, SR6, R1 and R12 (Figure 1). Blood samples were immediately centrifuged at 1100 g and plasma and serum aliquots frozen and stored at −80°C and used for subsequent adrenocorticotropic hormone (ACTH) and testosterone determination. ACTH and testosterone concentrations were assayed in duplicate by ELISA kit provided by IBL (IBL International, Hamburg, Germany). The minimum detectable concentration was 0.22 pg/mL and 0.24 nmol/L respectively. The intra- and inter-variability coefficients were: 6.7 and 7.1 for ACTH, and 5.4 and 4.2 for testosterone.

### Experimental Design

#### Pre-Experimental Procedure

During an 8 days period preceding the sleep restriction period, subjects were instructed to maintain a regular sleep–wake cycle with 8 h in bed (bedtime hour at 11 p.m. and wake-up time at 7 a.m.). All subjects wore a wrist activity monitor and completed a sleep–wake diary during this control period. The timing of the nocturnal sleep was not allowed to deviate from more than 1 h from the scheduled sleep periods of the laboratory (from 11:00 p.m. to 7:00 a.m.). Compliance with these instructions was verified by inspection of the rest–activity plots and the sleep–wake diaries.

#### Experimental Design (Figure 1)

During this experimental procedure each subjects have spent 14 complete days in-residence in the laboratory. A “*complete day*” is defined here as the time spent from the beginning of scheduled sleep period to the beginning of the next. Subjects were housed individually in a temperature-controlled bedroom (23 ± 1°C) at the Hotel-Dieu AP-HP Hospital (Paris, France). Laboratory illumination was maintained at 150–200 lux during the entire experimental period. Subjects were instructed to arrive at the sleep laboratory on mid-afternoon before the first day (B0). Nocturnal sleep was evaluated during 12 consecutive nights. The first two complete nights were used respectively as habituation and baseline ones (BN1 and BN2). On following days, subjects were sleep restricted during 7 days. They were allowed spending 4 h in bed for each night (SRN1 to SRN7: 4 h of TIB from 2:00 to 6:00 a.m., Figure 1). After 7 days of sleep restriction, subjects were then allowed spending 8 h of TIB as during baseline (RN1 to RN3: from 11:00 p.m. to 7:00 a.m.). Following these 12 complete days in-residence in the sleep laboratory, subjects were allowed to come back home and were instructed to wear a wrist activity monitor and to complete a sleep–wake diary during this 8 days period. Subjects came back to the sleep laboratory for two complete days with 8 h of TIB (from 11:00 p.m. to 7:00 a.m., Figure 1). Subjects were allowed to leave the sleep laboratory at the end of these two complete days (R12 at 8:00 p.m.). Throughout all these experimental stages, subjects were not permitted any other time in bed or opportunity for sleeping.

Subjects completed questionnaires (KSS) four times a day at baseline (B2), sleep restriction (SR1, SR4 and SR7) and during the recovery period days (R3 and R13). They completed visuo-motor, sustained attention tasks and executive tests (Go-NoGo and a 2-choice reaction time task) during a randomized testing session (lasting around 35 min) on morning (8:30–9:05 a.m.) and evening (6:30–7:05 p.m.) sessions of test days (i.e., at baseline: B2; during the 7 days sleep restriction period: SR1, SR4, SR7 and during the sleep recovery period: R2, R3 and R13, Figure 1).

### Statistical Analysis

All statistical analyses were conducted using statistical software (Statistica 8.0; StatSoft^®^). All data are presented as means ±standard error of the mean (SE). All value’s distributions were tested for their normality (Kolmogorov-Smirnov and Liliefors tests). If the value’s distribution was normal, then we used one or two ways analysis of variance (ANOVA) analyses with repeated measures. For *post hoc* analyses we essentially used Newman-Keuls (NK), Duncan (D) or LSD Tests (L). In contrary cases, we used one way non-parametric ANOVA test with repeated measures (ANOVA of Friedman and Wilcoxon test for* post hoc* comparison: W) or with no repeated measures (Kruskall-Wallis test: KW). Finally, we tested the correlative links between the most interesting and significant variables of cognitive tests and others behavioral (Age, MWT) and biological variables.

## Results

### Main Effects of Chronic Sleep Restriction

#### Time in Bed, WASO, Sleep Efficiency and TST

**Figure 2:**
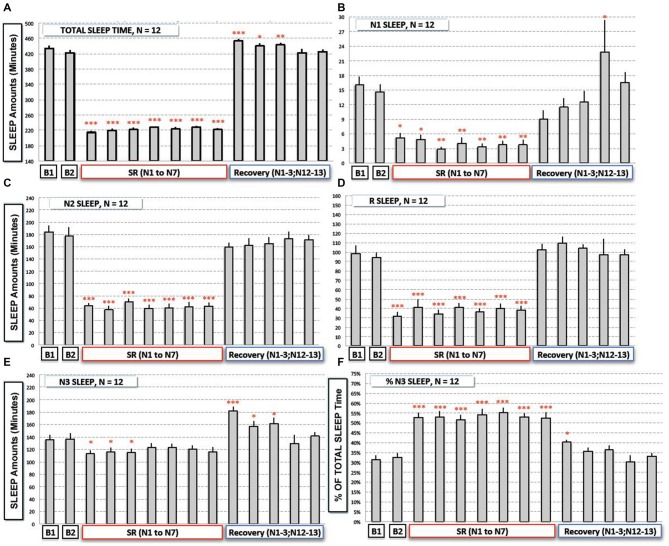
**Total and sleep stages (N1, N2, N3 and REM Sleep) durations during baseline (B1, B2), the sleep restriction (SRN1 to SRN7) and the following sleep recovery period (RN1–RN3, RN12 and RN13). (A)** Total sleep time, **(B)** N1 sleep, **(C,D)** N2 and REM sleep, **(E,F)** N3 sleep and proportion of N3 sleep (% of total sleep time) are represented (gray bars) as mean of amounts in min (or % for **F**; ± SEM) for the 12 subjects. Significant differences compared to baseline values (B2): *p < 0.05; **p < 0.01; ***p < 0.001.

Logically amounts of TIB and WASO were significantly reduced during the sleep restriction week (respectively, TIB: 480 ± 0 min vs. 239.9 ± 0.11 min and WASO: 21.6 ± 2.67 min vs. 5.6 ± 0.64 min; *F*_(13,143)_ = 6.39; *p* < 10^−6^). Conversely sleep efficiency was significantly increased during the sleep restriction week (89 ± 0.9% vs. 93 ± 0.5%; *F*_(13,143)_ = 5.36; *p* < 10^−6^). During the two baseline nights (B1 and B2, 8 h of TIB from 11:00 p.m. to 7:00 a.m.), subjects slept respectively 434.8 ± 2.1 min and 422.42 ± 2.1 min and these amounts were not significantly different from each other (*t*: 1.88; *p* > 0.086). Logically, TST amounts were significantly modified with restriction/recovery periods (*F*_(13,143)_ = 731.79; *p* < 10^−6^) with respectively a significant reduction of TST during the sleep restriction period and a significant increase of TST during the first three recovery nights (Figure 2A).

#### Amounts of the Different Sleep Stages (Figures 2B–F)

Results showed a significant time experiment effect on N1 amounts (*F*_(13,143)_ = 9.08; *p* < 10^−6^), N2 amounts *F*_(13,143)_ = 63.22; *p* < 10^−6^), N3 amounts *F*_(13,143)_ = 8.299; *p* < 10^−6^), and REM sleep amounts (*F*_(13,143)_ = 28.47; *p* < 10^−6^). More precisely we observed a significant reduction of N1, N2 and REM sleep amounts during the sleep restriction period (Figures 2B–D). A significant reduction of N3 sleep amount was also observed but to a lesser extent (Figure 2E). During sleep recovery nights, there was only a significant increase of N3 sleep during the first three sleep recovery nights (Figure 2E).

Despite the fact that total amount of sleep is reduced by a factor of two during the sleep restriction period, amounts of N3 sleep stage were only reduced by a factor of 1.15 (a mean of 118 min across the seven nights of sleep restriction compared to 136 min at B2) whereas amounts of other sleep stages were reduced by a higher factor (3.75 for N1, 2.8 for N2 and 2.5 for REM sleep). As a logical consequence, there was a significant sleep restriction/recovery effect on the proportion (% of TST) of N3 sleep (*F*_(13,143)_ = 23.55; *p* < 10^−6^), that was significantly higher during all the nights of the sleep restriction period (a mean proportion of 53.1% instead of 32.4% at B2; Figure 2F) and during the first night of sleep recovery (Figure 2F).

#### Subjective Sleepiness (KSS) and Capacity of Staying Awake (MWT; Table 1)

We observed a significant effect of the sleep restriction period (*F*_(5,55)_ = 8.58; *p* < 10^–5^). KSS scores were significantly higher after 4 and 7 days of sleep restriction compared to baseline’s one (D: *p* < 0.001 and *p* < 0.001 respectively for SR4 and SR7 compared to B2; Table 1). No significant time of day effect was observed for KSS scores completed during morning and evening periods (*F*_(1,22)_ = 1.02; *p* > 0.32) but we observed a significant sleep restriction period × time of day interaction (*F*_(5,110)_ = 2.38; *p* < 0.05). *Post hoc* analyses showed a significant increase of morning KSS scores during the sleep restriction period and no significant increase for evening ones (Morning score: D: *p* < 0.001 and *p* < 0.001 respectively for SR4 and SR7 compared to B2 and Evening score: D: *p* > 0.28 and *p* > 0.06 respectively for SR4 and SR7 compared to B2; Table 1).

**Table 1:** **Subjective sleepiness (KSS score) and capacity to stay awake (MWT Latencies) during baseline (B2), the sleep restriction period (SR1, SR4 and SR7) and the following period of sleep recovery (R3 and R13)**.

KSS Score/MWT Latencies	Baseline: B2	Restriction: SR1	Restriction: SR4	Restriction: SR7	Recovery: R3	Recovery: R13
Daily score	3.22 ± 0.31	3.82 ± 0.32	**4.66 ± 0.31**^###^	**4.73 ± 0.43**^###^	2.85 ± 0.42	2.92 ± 0.40
Morning score (10 am)	3.33 ± 0.47	4.25 ± 0.45	**5.38 ± 0.32**^###^	**5.38 ± 0.56**^###^	2.50 ± 0.40	2.67 ± 0.38
Evening score (4 pm)	3.25 ± 0.35	3.75 ± 0.39	4.17 ± 0.58	4.00 ± 0.35	2.58 ± 0.47	2.83 ± 0.47
Daily latencies	38.80 ± 0.73	39.39 ± 0.58	**34.29 ± 2.75**^#^	**28.78 ± 3.38**^#^	36.73 ± 1.75	38.52 ± 0.75
Morning latencies (10 am)	39.21 ± 0.79	39.04 ± 0.96	32.13 ± 4.14	**31.88 ± 3.75**^#^	38.17 ± 0.97	37.50 ± 1.56
Evening latencies (4 pm)	36.00 ± 2.92	39.88 ± 0.13	34.19 ± 2.78	28.92 ± 3.63	36.54 ± 2.52	38.42 ± 1.37

*KSS score and MWT latencies are represented as the mean (± SEM) in daily (all sessions), in morning (10:00 am) and in evening sessions (4:00 pm) for the 12 subjects. Significant differences compared to baseline value (B2): ^#^p < 0.05; ^###^p < 0.001*.

A significant time of experiment effect was observed for daily latencies to fall asleep ($\chi_{\left(12,5\right)}^{2}$ = 21.03, *p* < 0.001). These daily sleep latencies (latency to the first sleep epochs after light off) were significantly shorter during the sleep restriction period and more precisely after almost four nights of sleep restriction and not after one night of sleep restriction or during recovery nights (RN3 and RN13, Table 1). We observed for morning and evening sessions, a significant effect of the sleep restriction period ($\chi_{\left(12,5\right)}^{2}$ = 10.83, *p* = 0.054 and $\chi_{\left(12,5\right)}^{2}$ = 12.14, *p* < 0.05). Morning latencies were significantly shorter after almost seven nights of sleep restriction (W: SR7, *p* < 0.05) and no significant changes were observed for evening ones (Table 1).

#### Visuo-Motor Pursuit Task

For both deviations (horizontal and vertical measured as distance from center of the screen, RMS) and loss of control parameters, no significant time experiment effect was observed (respectively *F*_(6,66)_ = 0.767, *p* > 0.59, and $\chi_{\left(12,6\right)}^{2}$ = 10.40, *p* > 0.10, data not shown).

#### Simple Reaction Time Task (SRTT) and Go-NoGo Task (Figures 3A–D)

**Figure 3:**
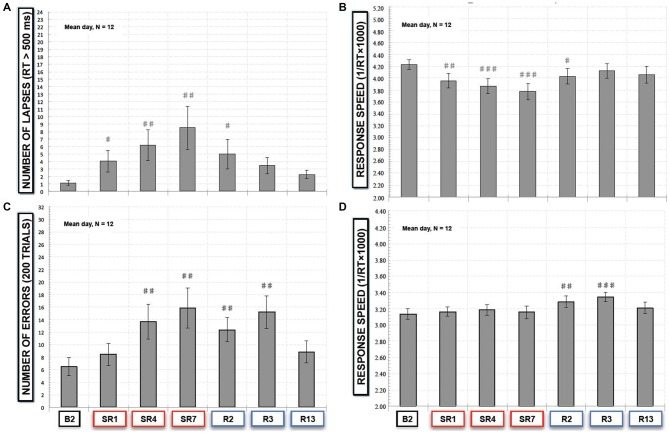
**(A)** Number of lapses responses (*RT* > 500 ms) and **(B)** response speed in the simple reaction time task (SRTT) task and **(C)** number of errors (“NoGo responses”) and **(D)** response speed of “Go” trials in the Go-NoGo task. All these variables are represented as the mean (± SEM) of the 12 subjects that performed at baseline (B2), during the sleep restriction period (SR1, SR4 and SR7), and the following period of sleep recovery (R2, R3 and R13). Significant differences compared to baseline values (B2): ^#^*p* < 0.05; ^##^*p* < 0.01; ^###^*p* < 0.001.

In the SRTT task, we did not find a significant sleep restriction/recovery effect on the mean number of anticipation responses (*F*_(6,66)_ = 0.40; *p* > 0.87). Consequently the number of lapses, is directly linked to the number of correct responses (150 ms < latency response < 500 ms), that was significantly modified by the sleep restriction/recovery experiment ($\chi_{\left(12,6\right)}^{2}$ = 24.39, *p* < 0.001) with a significant increase of the number of lapses during the sleep restriction period in comparison with baseline values (*p* < 0.01 compared to B2, Figure 3A) and after two nights of sleep recovery (*p* < 0.01, Figure 3A). No significant differences were observed after the third night of sleep recovery. Concerning the speed of response, we observed a time experiment effect (*F*_(6,66)_ = 7.14; *p* < 0.001, Figure 3B). During the sleep restriction period subjects significantly performed slower during the 1st, the 4th and the 7th days of sleep restriction and R2 compared to B2 (*p* < 0.001 and *p* < 0.05 respectively, Figure 3B).

In the executive Go-NoGo task, we observed a significant sleep restriction effect on the number of errors (commission errors with no-go responses; $\chi_{\left(12,6\right)}^{2}$ = 35.56, *p* < 10^−5^). During the sleep restriction period, there was a significant increase of errors that stayed significantly higher during the 2nd and the 3rd night of sleep recovery in comparison with baseline values (*p* < 0.01, Figure 3C). We also observed a significant sleep restriction/recovery effect on the speed of good responses (*F*_(6,66)_ = 4.81; *p* < 0.001, Figure 3D). But this was an increase in response speed that was only observed during the 2nd and the 3rd night of sleep recovery (Figure 3D).

#### Sensory Decision-Making (Simon) Task

For this sensory-motor decision-making task we did not find any significant sleep restriction period effect on the number of errors for all trials (*F*_(6,66)_ = 1.63; *p* > 0.15). This lack of effect was also observed for both spatial compatible (*F*_(6,66)_ = 1.54; *p* > 0.17) and spatial incompatible trials (*F*_(6,66)_ = 1.73; *p* > 0.12). We observed a significant time of experiment effect on the speed of correct responses for all trials (*F*_(6,66)_ = 8.18; *p* < 10^−5^). More precisely, there was a significant increase of the response speed during the 2nd, the 3rd and the 13th day of sleep recovery (*p* < 0.01 and *p* < 0.001, respectively) for both spatially compatible trials (*F*_(6,66)_ = 7.26; *p* < 10^−5^; *p* < 0.01; *p* < 0.001 and *p* < 0.01) and spatially incompatible trials (*F*_(6,66)_ = 6.92; *p* < 10^−5^; *p* < 0.01 and *p* < 0.001).

### Modulation of Sleep Restriction’s Effects by the Time of Day

#### Morning and Evening Differences in SRTT and Go-NoGo Performances (Figures 4A–D)

**Figure 4:**
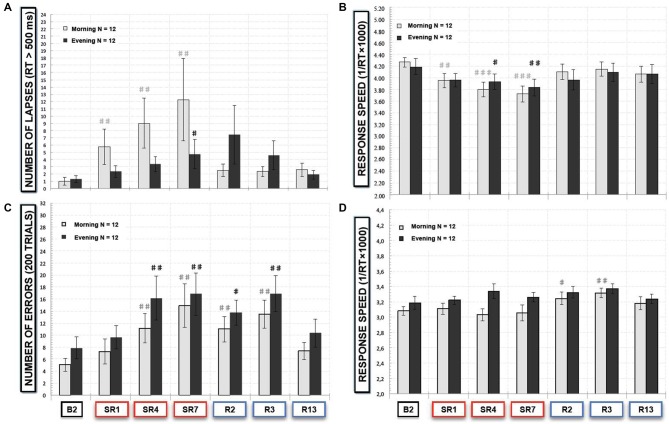
**(A)** Number of lapses responses (*RT* > 500 ms) and **(B)** response speed in the SRTT task and **(C)** number of errors (“NoGo responses”) and **(D)** response speed of “Go” trials in the Go-NoGo task, that subjects (*N* = 12) performed in morning (8:30–9:30 am, gray bars) and evening (6:30–7:30 pm, black bars) at baseline (B2), during the sleep restriction period (SR1, SR4 and SR7) and the following period of sleep recovery (R2, R3 and R13). All these variables are represented as the mean (± SEM) of the 12 subjects that performed at baseline (B2), during the sleep restriction period (SR1, SR4 and SR7), and the following period of sleep recovery (R2, R3 and R13). Significant differences compared to baseline values (B2): ^#^*p* < 0.05; ^##^*p* < 0.01; ^###^*p* < 0.001.

In the SRTT task, we observed a significant sleep restriction/recovery effect both for morning ($\chi_{\left(12,6\right)}^{2}$ = 20.75, *p* < 0.01) and evening sessions ($\chi_{\left(12,6\right)}^{2}$ = 13.56, *p* < 0.05). But we observed a significant increase of lapses compared to B2 after the 1st night and during all the sleep restriction period in morning session tests and only after SR7 in evening test sessions (Figure 4A). As regards of the speed of correct responses, we did not find any time of day effect (*F*_(6,66)_ = 0.85; *p* > 0.52). But we observed a faster and larger decrease of correct responses in speed with a significant decrease compared to B2 after the SR1 in morning session tests whereas this decrease was significant after the SR4 in evening session tests (Figure 4B).

In the executive Go-NoGo task, we observed a significant effect for both morning ($\chi_{\left(12,6\right)}^{2}$ = 36.10, *p* < 10^−5^) and evening sessions ($\chi_{\left(12,6\right)}^{2}$ = 22.77, *p* < 0.001). Numbers of errors were significantly increased with the sleep restriction/recovery period in a similar manner for morning session tests and for evening session ones (Figure 4C). Concerning response speed of correct responses (“Go” trials), we did not show any time of day effect (*F*_(6,132)_ = 1.95; *p* > 0.07). But we observed differences between morning and evening test sessions with a significant increase in the response speed during the 2nd and the 3rd day of sleep recovery for morning test sessions (Figure 4D).

#### Morning and Evening Differences in Simon Task

We did not find any time of day (morning/evening) effect on the number of errors for all trials (*F*_(6,132)_ = 0.79; *p* > 0.57) and also for both spatial compatible (*F*_(6,132)_ = 0.42; *p* > 0.85) and spatial incompatible trials (*F*_(6,132)_ = 1.49; *p* > 0.18). Concerning speed of correct responses, we neither observed a significant time of day effect for all trials (*F*_(6,132)_ = 0.301; *p* > 0.93) nor for both spatial compatible (*F*_(6,132)_ = 0.112; *p* > 0.99) and spatial incompatible trials (*F*_(6,132)_ = 0.75; *p* > 0.60).

#### Biological Parameters

##### Circulating Concentrations of Adrenocorticotropic Hormone (ACTH) and Testosterone (Table 2)

We did not observe a significant effect of the sleep restriction period on morning ACTH concentrations ($\chi_{\left(12,3\right)}^{2}$ = 3.50, *p* > 0.32). We, however, observed a significant effect on morning (8:00 a.m.) concentrations of testosterone (*F*_(3,33)_ = 9.35; *p* < 0.001) with a significant reduction of testosterone concentrations after six sleep-restricted nights (D: SR6: *p* < 0.01 compared to B1, Table 2). No significant differences with baseline values were observed at R1 and R12 (Table 2).

**Table 2:** **Morning circulating concentrations of testosterone and ACTH measured at baseline (B1), after six nights of sleep restriction (SR6) and after 1 or 12 sleep recovery nights (R1 and R12)**.

Circulating Concentrations	Baseline: B1	Restriction: SR6	Recovery: R1	Recovery: R12
Testosterone (nmol/L)	17.17 ± 1.51	**13.89 ± 1.07^##^**	15.86 ± 1.01	19.15 ± 1.72
ACTH (pg/mL)	50.40 ± 6.14	38.20 ± 6.78	48.01 ± 5.66	49.65 ± 8.17

*Concentrations are represented as mean ± SEM (n = 12). Significant differences compared to baseline values (B1): ^##^p < 0.01*.

##### Salivary Concentrations of Cortisol and α-Amylase (sAA; Table 3)

We observed a significant effect of the sleep restriction period for both cortisol and alpha-amylase concentrations (respectively *F*_(3,33)_ = 3.62; *p* < 0.05 and $\chi_{\left(12,3\right)}^{2}$ = 13.89, *p* < 0.01). Compared to baseline values (B2), concentrations of cortisol and α-amylase in saliva were significantly lower after seven nights of sleep restriction and stayed lower until almost two nights of sleep recovery for cortisol (D: *p* < 0.05 SR7 and R2; Table 3) and stayed lower after 13 sleep recovery nights compared to baseline values for α-amylase concentrations (W: *p* < 0.01 for SR7 and R2 and *p* < 0.05 for R13; Table 3).

**Table 3:** **Morning salivary cortisol and α-amylase concentrations measured at baseline (B2), after seven nights of sleep restriction (SR7) and after 2 or 13 sleep recovery nights (R2 and R13)**.

Saliva Concentrations	Baseline: B2	Restriction: SR7	Recovery: R2	Recovery: R13
Cortisol (nmol/L)	8.97 ± 1.45	**6.00 ± 0.97^#^**	**5.41 ± 0.72^#^**	8.25 ± 0.74
α-AMYLASE (U/mL)	48.66 ± 9.90	**27.20 ± 3.79^##^**	**29.50 ± 4.53^##^**	**35.16 ± 5.96^#^**

*Concentrations are represented as mean ± SEM (n = 12). Significant differences compared to baseline values (B2): ^#^p < 0.05; ^##^p < 0.01*.

### Relationships Between Cognitive Variables, MWT Latencies, Age of Subjects and Biological Markers

We looked at relationship between main cognitive test variables (number of lapses and mean speed for SRTT and morning or evening errors of commission for Go-NoGo), during baseline, at the end of the seven nights of sleep restriction and after three-recovery nights of sleep, and variables related to the capacity of subjects to stay awake, such as daily, morning or evening MWT latencies, their age and biological markers modified by the sleep restriction period such as testosterone or ACTH circulating concentrations and salivary cortisol or α-amylase.

#### Simple Reaction Time Task (Figure 5A)

**Figure 5:**
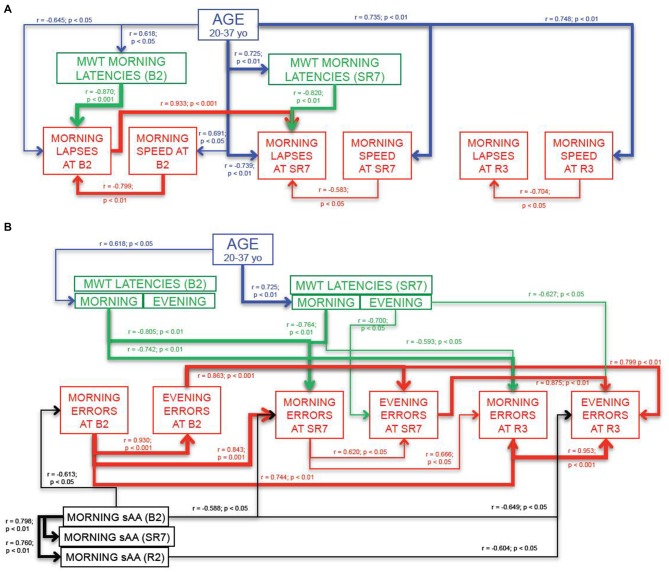
**Main variables that are correlated with SRTT (A) and Go-NoGo variables (B).** Each line represents a significant correlation (*r* coefficient and *p* values written closed to the lines) between two variables. The strength of the relationship is symbolized by the thickness of the arrow. Thin arrows represent weak but significant correlations (*p* < 0.05) and thicker arrows symbolize more significant ones (*p* < 0.01 and *p* < 0.001). Blue lines represent correlations between test (or MWT latencies) variables and age of subjects. Green lines represent correlations between test variables and MWT latencies. Red lines represent correlations between variables inside each cognitive test; SRTT **(A)** and Go-NoGo task **(B)**. Black lines represent correlations between test variables and morning α-amylase concentrations in saliva samples.

Results showed that for sustained attention, morning lapses and morning speed at the end of the sleep restriction period (SR7) were respectively negatively (*r* = −0.739; *p* < 0.01) and positively (*r* = 0.735; *p* < 0.01) correlated with age of subjects (Figure 5A). Only morning lapses at SR7 were negatively correlated with both MWT morning latencies at SR7 (*r* = −0.820; *p* < 0.01 for SR7) and with morning speed at SR7 (*r* = −0.583; *p* < 0.05; Figure 5A).

Interestingly, these correlations were also observed during baseline (B2) but with a weaker way, for instance with age (Figure 5A). Moreover morning lapses at SR7 were strongly and positively correlated with morning lapses at B2 (*r* = 0.933; *p* < 0.001; Figure 5A). During recovery (R3), only morning speed was significantly and positively correlated with age of subjects (*r* = 0.748; *p* < 0.01) and this behavioral variable was negatively correlated with morning lapses at R3 (*r* = −0.704; *p* < 0.05; Figure 5A).

No significant relationships were observed between evening lapses, evening speed, MWT latencies and age of the subjects.

#### Go-NoGo Task (Figure 5B)

Our results showed that for inhibition, morning errors at the end of the sleep restriction period (SR7) were positively and strongly correlated with morning errors at baseline (B2: *r* = 0.843; *p* < 0.001) and evening errors at SR7 were positively and strongly correlated with those at B2 (*r* = 0.863; *p* < 0.001). Morning and evening errors after three-recovery nights of sleep (R3) were positively, significantly and respectively correlated with both morning errors at B2 (*r* = 0.744; *p* < 0.01) and at SR7 (*r* = 0.666; *p* < 0.05) and with evening errors at the same time (B2: *r* = 0.799; *p* < 0.01; SR7: *r* = 0.875; *p* < 0.01; Figure 5B). Moreover evening errors were strongly correlated with morning ones at B2 (*r* = 0.930; *p* < 0.001) and at R3 (*r* = 0.953; *p* < 0.001**)** and to a weaker way at SR7 (*r* = 0.620; *p* < 0.05; Figure 5B).

Morning errors at SR7 and R3 were negatively correlated with both MWT morning latencies at B2 (respectively *r* = −0.805; *p* < 0.01 for SR7 and *r* = −0.742; *p* < 0.01 for R3) and at SR7 (respectively *r* = −0.764; *p* < 0.01 for SR7 and *r* = −0.593; *p* < 0.05 for R3; Figure 5B). Besides, evening errors at SR7 and at R3 were negatively correlated with MWT evening latencies at SR7 (respectively *r* = −0.700; *p* < 0.05 for SR7 and *r* = −0.627; *p* < 0.05 for R3; Figure 5B).

Finally, we also observed negative correlations between morning errors at B2, at SR7 and evening errors at R3 with B2 morning concentrations of α-amylase (respectively; *r* = −0.613; *p* < 0.05; *r* = −0.588; *p* < 0.05 and *r* = −0.649; *p* < 0.05; Figure 5B). Evening errors at recovery (R3) were also negatively correlated with morning concentrations of α-amylase at R2 (*r* = −0.604; *p* < 0.05; Figure 5B).

No significant relationships were observed between morning or evening errors and age of the subjects.

## Discussion

The two main findings of this sleep restriction’s experiment are, in spite of a logical reduction of total sleep time and sleep stages (N1, N2, N3 and REM sleep) and a subjective increase of sleepiness for all subjects: (i) a differential kinetic in deterioration and recovery of three types of cognitive processes (sustained attention, motor inhibition and sensory-motor decision-making); and (ii) a differential modulation of these effects by time of day (morning vs. evening), subject’s capacity to stay awake, and age. More generally, this sleep restriction experiment highlights the hypothesis that: (1) under a situation of CSR, cognitive capacities are not equally disturbed and restored; and that (2) individual vulnerability to CSR are modulated by baseline’s capabilities and biological changes that would implicate the stress-related activity of the HPA axis, the SAM system and the anabolic hormone testosterone and potentially subject’s brain maturation degree.

More precisely, this study shows, as expected, that under a chronic lack of sleep (4 h of TIB instead of 8), all subjects spent immediately (1st night) less time in all sleep stages (N1, N2, N3 and REM sleep) and spent proportionally more time in deep NREM sleep (around 50–55% of total sleep time) and less than a third of time in light sleep (N2 and less than 2% in N1 sleep stage) and less than a fifth in REM sleep. This sleep restriction’s week is immediately followed by a sleep rebound during the 3rd recovery night with a significant increase of both N3 sleep amounts and proportion. These results are in accordance with previous ones showing significant reductions of total sleep durations, as well as light and REM sleep duration, during several nights of restricted sleep (Belenky et al., 2003; Van Dongen et al., 2003; Rupp et al., 2009; Banks et al., 2010; Lo et al., 2012). Concerning NREM sleep, we observed a significant decrease of NREM sleep amounts during the first three nights of sleep restriction followed by three nights with no significant reduction and a final 7th day with a slight decrease that was barely significant. This observation seems to contradict previous works showing no NREM sleep reduction (Belenky et al., 2003; Van Dongen et al., 2003). However, a more thorough analysis of our results indicated that this decrease of NREM sleep amounts concerns only the first three nights of sleep restriction and represents a partial decrease of 15%, two results that are consistent with two previous works (Axelsson et al., 2008; Banks et al., 2010).

Under such sleep restriction conditions, we observed that subjects felt sleepy from the 4th day until the last day and this subjective complaint seems to rapidly ceil at a score of 5. These results are in line with those of previous studies (Belenky et al., 2003; Van Dongen et al., 2003; Kobayashi et al., 2007; Lo et al., 2012; Philip et al., 2012). In parallel, our results demonstrate that a week of severe sleep restriction (4 h of TIB) is both responsible for sustained attention deficits, with a decrease of response speed and an increase in the number of lapses, and executive disorders with an increase in the numbers of errors (“No-Go” trials) while sleepy subjects were still able to perform a sensori-motor coordination task. These results, once again, agree with previous studies showing that a sleep-restricted week is responsible for sustained attention deficits with both a decrease of response speed and an increase in the number of lapses in a linear way (Belenky et al., 2003; Van Dongen et al., 2003; Rupp et al., 2009; Philip et al., 2012) and for executive processes impairments (Lo et al., 2012; Sagaspe et al., 2012). Moreover, Bratzke and colleagues observed, across 40 h of continuous wakefulness under controlled environmental and behavioral conditions, that interference and facilitation in the Simon task (sensori-motor decision-making task) did not show any significant variation (Bratzke et al., 2012). Interestingly, we show that the kinetic of failures and recovery of such cognitive capabilities are different. Indeed, nearly half of the deficits in sustained attention (increase of lapses and decrease of response speed) were observed after one sleep-restricted night whereas deficit was observed neither for executive processes (i.e., motor inhibition) nor for sensory-motor decision-making capacities. Furthermore, with young and healthy subjects (from 20 to 37 years old), we observed a differential recovery kinetic of such cognitive capacities with at least two sleep-recovery nights (8 h of TIB) to recover from sustained attention deficits and more than three sleep-recovery nights to fully recover from motor inhibition capabilities. Our observations, one more time, are in line with those of Lo et al. (2012) study showing that sustained attention capacity was more rapidly affected than working memory across 7 days of 6 h sleep-restricted nights in healthy young individuals (Lo et al., 2012) and with those studies describing that some neurobehavioral functions may not return to baseline following up to 1–2 recovery sleep periods limited to 8 h TIB (Belenky et al., 2003; Axelsson et al., 2008; Banks et al., 2010). But here we show that this recovery kinetic is different for sustained attention compared to executive processes (i.e., motor inhibition). Meta-analyses of studies focusing on cognitive effects of TSD indicated that attention as well as working memory were the cognitive processes the more affected by TSD (Lim and Dinges, 2010; Reynolds and Banks, 2010; Ma et al., 2015). More interestingly, a recent meta-analysis of studies focusing on cognitive effects of CSR or disruption have pointed out that complex cognitive task performance may not be impacted by restricted sleep as severely as simple cognitive task performance (Wickens et al., 2015).

The most interesting findings of this work are the differential modulation in deficits and recovery of sustained attention and executive (i.e., motor inhibition) processes with: (1) the time of day (morning vs. evening periods); (2) subject’s capacity to stay awake; and (3) subject’s age. Concerning the first point, while morning SRTT lapses and speed respectively increased and decreased more rapidly (as early as the 1st sleep-restricted night) compared to the same evening variables (respectively the 7th and the 4th sleep-restricted nights), we did not observe such time of day differences with an executive (Go/Nogo) task in which we found a similar kinetic of failures and recovery. We thus observed, in a chronic sleep restricted situation (seven nights with 4 h of TIB instead of 8), a differential modulation of deficits and recovery of these two different cognitive processes by the time of day. This assessment is in accordance with previous studies showing a time window of vulnerability at morning hours (around 5–9 a.m.) for sustained attention processes in healthy young subjects submitted to several sleep-restricted nights (Cohen et al., 2010; Mollicone et al., 2010; Lo et al., 2012) and also with earlier work showing no circadian effects for executive processes in a TSD protocol (Bratzke et al., 2012; Sagaspe et al., 2012). One possible explanation come from Drummond and colleagues who have suggested, in a TSD experiment with subjects undertaking a Go-NoGo task, that TSD differentially alters attention for automatic responding and response withholding (Drummond et al., 2006). It was established that automatic responding and withholding a response may engage different brain regions with automatic responding typically activating sustained attention with an activation of the right dorsolateral prefrontal cortex (PFC; Culham and Kanwisher, 2001; Yamasaki et al., 2002) whereas the right ventro-lateral part of the frontal cortex is engaged in response inhibition (Aron et al., 2004). Moreover it is well known that prolonged wakefulness is responsible for a significant reduction of cerebral metabolism (Wu et al., 1991; Thomas et al., 2000) but with a significant and specific metabolic reduction of the fronto-parietal network (for review see Chee and Chuah, 2008; Verweij et al., 2014; Ma et al., 2015), dedicated to attention (Bisley and Goldberg, 2010) and notably to sustained attention (Drummond et al., 2005a). We thus hypothesize that a chronic sleep debt will differentially disturb brain networks and the fronto-parietal network will be more rapidly affected compared to other cortical areas such as the ventral part of the prefrontal cortex implicated in executive processes.

For the two other interesting findings, namely a differential modulation of sustained attention and executive failures and recovery with age of subjects and their capacity to stay awake, we showed that subjects that are more vulnerable to sustained attention deficits under sleep restriction were the younger ones and this relationship was also observed to a lesser extent at baseline. Interestingly the number of lapses obtained at SR7 was negatively correlated with subject’s capacity to stay awake in sleepy conditions (MWT task at SR7), the latter positively correlated with age of the subjects. These two relationships were also observed with baseline values. Concerning inhibition process, our results pointed out that there is no more relationship between age of the subjects and their performance at baseline (B2) or at the end of the sleep restriction period (SR7). Only subject’s capacity to fight sleepiness (MWT latencies at SR7) was significantly and negatively correlated with their numbers of errors in a Go-NoGo task but independently from the time of day (i.e., morning MWT latencies were only correlated to morning errors and evening MWT latencies to evening ones). Finally subjects’ performances at SR7 and at R3 in the Go-NoGo task were strongly and positively correlated with their own performances at baseline both (and independently) for morning and evening test sessions. In our point of view, these findings are new but not surprising. Indeed, the ability to cognitively resist to the deleterious effects of TSD seems to be a trait-like phenomenon (Van Dongen et al., 2004; Chee and Tan, 2010; Rupp et al., 2012; Chua et al., 2014b; Xu et al., 2016) with potential genetic (Viola et al., 2007; Groeger et al., 2008; Goel and Dinges, 2012), neurobiological (Yeo et al., 2015) and/or psychological profiles (Killgore et al., 2007b). More recently, Chua et al.’s (2014a,b) apostrophe studies (2014) demonstrated that subjects more vulnerable to the deleterious effects of TSD on sustained attention capacities show slower and more variable time response and lapses when they are well rested (Chua et al., 2014a,b). We go one step further with this study, by demonstrating similar individual vulnerability in a chronic sleep-restricted situation but with a significant implication of both age and subject’s capacity to fight sleepiness on morning failures of sustained attention and only of subject’s capacity for executive ones. Our findings agree with a previous study showing that under a CSR (7 days with 3 h TIB), sustained attention of a younger subjects (29 years old) were more disturbed than an older one (49 years old; Bliese et al., 2006). Our findings also agree with previous studies showing that subjects engaged in and performing well on a PVT task showed greater cerebral responses within the fronto-parietal network (and also cortical and subcortical motor systems), known to be affected by TSD (Mu et al., 2005a,b) whereas slow reaction times and/or subjects performing bad, particularly after TSD, showed greater activity in the default mode network (Drummond et al., 2005b; Xu et al., 2016). Interestingly, Yeo et al. (2015) showed that more resilient individuals to sustained attention decline following TSD exhibit stronger whole brain signal during their rested-state and inversely functional connectivity between Brain Default and Attention networks in the rested state appears to be a marker for vulnerability. According to these authors, individual differences in the ability to recruit fronto-parietal circuits or deactivate parts of the Brain Default Network during task performance could be determinants of performance in the sleep-deprived state. We thus hypothesize that the modulating effect of age on increasing sustained attention failures under CSR could be due to a deficit in the ability of younger subjects to recruit such fronto-parietal networks, possibly through non matured frontal areas (Gogtay et al., 2004). This is in accordance with a previous study showing with subjects performing a simple speeded-processing task during fMRI scanning, that faster performers show greater neural activity in parietal regions and less frontal activity compared to slower performers (Rypma et al., 2006). Using regional-causality analysis this study also demonstrated that frontal regions exert more influence over other brain regions for slower performances in comparison to faster ones, suggesting that a critical determinant of individual performance differences is the efficiency of interactions between brain regions (Rypma et al., 2006). Concerning executive failures during CSR (SR7), sleep recovery (R3) and their significant and positive links with baseline performance, we hypothesize that such individual vulnerability is linked to individual baseline efficiency. Indeed previous studies have shown an increase of neural activity in PFC following sleep deprivation related to an increase of working memory load (Choo et al., 2005; Drummond et al., 2005b; Mu et al., 2005b). Thus we can imagine that individuals that were vulnerable in their capacity to inhibit their motor response at SR7 would be individuals that are already in a brain state that is not very active at baseline. This is in agreement with a previous study showing that individuals who are TSD-vulnerable for working memory performance presented a lower activation of their brain (especially frontal and parietal regions) at rested baseline compared to TSD-resilient subjects (Mu et al., 2005a).

Finally we showed: (i) a significant decrease of morning cortisol concentrations in saliva at SR7 and at R2 (after 2 sleep-recovery nights) with a return to baseline values at the end (R13); and (ii) a significant decrease of morning salivary alpha-amylase (sAA) concentrations at SR7, at R2 and also at R13. These biological results, especially for cortisol suggest that chronic lack of sleep exerts a deleterious effect on the HPA axis. It is well demonstrated that NREM sleep and HPA axis exert reciprocal inhibitory effects (Vgontzas et al., 1999; Steiger, 2002). Thus we hypothesize that our seven sleep-restricted nights has only slightly reduced NREM sleep amounts (only the first three nights) but above all has increased its percentage over total sleep time (from SRN1 to SRN7) thus contributing to a decrease of morning cortisol concentrations at SR7. After a second night of sleep recovery (RN2), increased amount of NREM sleep and recovery of its percentage may contribute to explain decreased morning cortisol concentration. This hypothesis is in accordance with results of a recent team’s article (Arnal et al., 2016) showing that TSD (24–34 h of wake) only decreases morning (7:00 a.m.) concentrations of plasmatic cortisol without modifying evening (5:00 p.m.) ones. Since this hormone is known to act as a stressor and alerting hormone for the body and the brain (Elder et al., 2014) and that frontal regions continue their maturation until 25–30 years old (Gogtay et al., 2004), we think that this would lead to a progressive morning time window of vulnerability of sustained attention with individual differences. Indeed, significant correlations between age of subjects and their sustained attention failures at SR7 (both for morning speed and lapses) and to a lesser extent at baseline (B2) let us think that under such sleep-restricted situations, brain of healthy and young subjects will not be affected in the same manner with a greater deleterious effect for younger men compared to older ones.

Morning number of errors in the Go-NoGo task at B2 and SR7 were significantly and negatively correlated with sAA concentration at B2, while evening errors at R3 were only significantly and negatively correlated with sAA concentration at B2 and R2. sAA concentration reflects blood levels of catecholamine, particularly norepinephrine (Chatterton et al., 1996) and thereby, sAA is considered as a useful tool for evaluating SAM activity (Walsh et al., 1999). There is scarce data on change of sAA concentration during experimental acute or chronic sleep deprivation. A blunted reactivity of sAA was observed when male subjects with chronic psychological stress were subjected to an acute mental stressor and this change was suggested to contribute to impairments in cognitive performance (Teixeira et al., 2015). After a mental stress event, changes in sAA were more remarkable than those in salivary cortisol (Strahler and Ziegert, 2015). In humans, the noradrenergic system was evidenced to modulate attentional processing and memory in a centrally mediated manner (Coull et al., 1999). Consistent with its role for attention, the medial prefrontal cortex is a target region for the noradrenergic system. This noradrenergic system has been recently demonstrated in humans to be involved in the modification of established associations during extinction learning and thus to play a role in behavioral flexibility, with medial PFC activation (Lissek et al., 2014). Considering our results, we argue that executive failures, most likely in the evening, would be related to the decrease of adrenergic responsiveness induced by CSR. Nevertheless, this laboratory experiment without an independent control group limits the possibilities to provide more detailed information concerning effects of a CSR on sleep parameters and cognitive performance and to generalize these results to everyday life and thus field studies are needed to confirm our findings.

## Conclusion

To our knowledge this is the first study to report a differential kinetic in failure and recovery of cognitive capacities of young and healthy men (20–37 years old) exposed to seven sleep-restricted nights depending on the cognitive process engaged (sustained attention or executive process such as inhibition), their age and their capacity to fight sleepiness, with some hypothesis for the biological mechanisms implicated. The present study confirms results of previous CSR’s experiments obtained with young subjects showing sustained attention failures (PVT task) with a time window of vulnerability during the morning period. Our work extends the comprehension that a chronic sleep debt does not equally affect cognitive processes of healthy subjects and that the time window of vulnerability for sustained attention processes does not exist for more executive functions.

From a practical point of view, this work provides new insights to study new potential and specific countermeasures to manage cognitive failures and inter-individual vulnerability due to CSR situations that exist in various professional areas (military, shift workers, long-distance drivers, health workers etc.). Further studies are needed to: (i) confirm a possible cognitive vulnerability to a CSR that would be related to age due to a lesser maturation of frontal cortical areas of the brain in younger subjects compared to older ones; (ii) examine if there is a specific implication of the corticotropic and adrenergic axis; and (iii) examine whether extending sleep with pharmacological aids has the same beneficial effects.

## Author Contributions

Authors have made substantial contributions to the following: Conception and design of the study: AR, CB, PVB, GD, MG, CD, PJA, FS, DL, MC. Acquisition of data: AR, LRP, CB, PVB, GD, MG, CYB, CD, PJA, FS. Analysis of data: AR, LRP, CB, PVB, MG, CD, MC. Interpretation of data: AR, DG, LRP, CB, CD, DL, MC. Writing the manuscript: AR, DGM, LRP, PVB, GD, CD, MC. Revisiting the manuscript: AR, DGM, CB, MG, CYB, PA, FS, DL, MC. Final approval of the version to be submitted: AR, DGM, LRP, CB, PVB, GD, MG, CYB, CD, PJA, FS, DL, MC. Agreement to be accountable for all aspects of the work in ensuring that questions related to the accuracy or integrity of any part of the work are appropriately investigated and resolved: AR, DGM, LRP, CB, PVB, GD, MG, CYB, CD, PJA, FS, DL, MC.

## Funding

Financial supports were provided by the *French General Directorate for Armament* (DGA, Department of Defense, Contract Number: PDH-1-SMO-2-505/12ca706).

## Conflict of Interest Statement

The authors declare that the research was conducted in the absence of any commercial or financial relationships that could be construed as a potential conflict of interest.

## Abbreviations

α-amylase: Alpha-amylase
MWT: Maintenance of Wakefulness Test
NREM Sleep: Non-Rapid Eye Movement Sleep
REM Sleep: Rapid Eye Movement Sleep
SRTT: Simple Reaction Time Task
TIB: Time In Bed
TSD: Total Sleep Deprivation
TST: Total Sleep Time
WASO: Wake After Sleep Onset.
